# Nested calcium dynamics support daily cell unity and diversity in the suprachiasmatic nuclei of free-behaving mice

**DOI:** 10.1093/pnasnexus/pgac112

**Published:** 2022-07-11

**Authors:** Lama El Cheikh Hussein, Pierre Fontanaud, Patrice Mollard, Xavier Bonnefont

**Affiliations:** Institut de Génomique Fonctionnelle, Université de Montpellier, CNRS, INSERM, IGF, 141 Rue de la Cardonille, F-34094 Montpellier, Cedex 5, France; BioCampus Montpellier, Université de Montpellier, CNRS, INSERM, 141 Rue de la Cardonille, F-34094 Montpellier, Cedex 5, France; Institut de Génomique Fonctionnelle, Université de Montpellier, CNRS, INSERM, IGF, 141 Rue de la Cardonille, F-34094 Montpellier, Cedex 5, France; BioCampus Montpellier, Université de Montpellier, CNRS, INSERM, 141 Rue de la Cardonille, F-34094 Montpellier, Cedex 5, France; Institut de Génomique Fonctionnelle, Université de Montpellier, CNRS, INSERM, IGF, 141 Rue de la Cardonille, F-34094 Montpellier, Cedex 5, France; BioCampus Montpellier, Université de Montpellier, CNRS, INSERM, 141 Rue de la Cardonille, F-34094 Montpellier, Cedex 5, France; Institut de Génomique Fonctionnelle, Université de Montpellier, CNRS, INSERM, IGF, 141 Rue de la Cardonille, F-34094 Montpellier, Cedex 5, France

**Keywords:** circadian rhythms, in vivo imaging, calcium signaling

## Abstract

The suprachiasmatic nuclei (SCN) of the anterior hypothalamus host the circadian pacemaker that synchronizes mammalian rhythms with the day–night cycle. SCN neurons are intrinsically rhythmic, thanks to a conserved cell-autonomous clock mechanism. In addition, circuit-level emergent properties confer a unique degree of precision and robustness to SCN neuronal rhythmicity. However, the multicellular functional organization of the SCN is not yet fully understood. Indeed, although SCN neurons are well-coordinated, experimental evidences indicate that some neurons oscillate out of phase in SCN explants, and possibly to a larger extent in vivo. Here, to tackle this issue we used microendoscopic Ca^2+^_i_ imaging and investigated SCN rhythmicity at a single cell resolution in free-behaving mice. We found that SCN neurons in vivo exhibited fast Ca^2+^_i_ spikes superimposed upon slow changes in baseline Ca^2+^_i_ levels. Both spikes and baseline followed a time-of-day modulation in many neurons, but independently from each other. Daily rhythms in basal Ca^2+^_i_ were highly coordinated, while spike activity from the same neurons peaked at multiple times of the light cycle, and unveiled clock-independent coactivity in neuron subsets. Hence, fast Ca^2+^_i_ spikes and slow changes in baseline Ca^2+^_i_ levels highlighted how multiple individual activity patterns could articulate within the temporal unity of the SCN cell network in vivo, and provided support for a multiplex neuronal code in the circadian pacemaker.

Significance Statement Living organisms have evolved exquisite mechanisms to anticipate and cope with changes imposed by the day–night cycle in their environment. The molecular machinery that sets the physiology of individual cells on a 24-hour period has now been deciphered. However, it is still unclear how multiple and diverse clock cells tick all together in the suprachiasmatic nuclei (SCN) of the hypothalamus, where the mammalian circadian pacemaker resides. In this study, we performed deep-brain calcium imaging to investigate daily rhythmicity at a single-cell resolution in free-behaving mice. We found a combination of two independent calcium dynamics in individual neurons of the SCN, which provides support for encoding both temporal unity and functional diversity in this composite neural system.

## Introduction

Circadian clocks rely on a conserved molecular mechanism expressed in virtually all body cells (1). In the suprachiasmatic nuclei (SCN) of the anterior hypothalamus, circuit-level interactions confer a unique degree of precision and robustness to circadian neuronal rhythms, which turns cell-autonomous oscillators into the central pacemaker that synchronizes mammalian physiology and behaviors with the day–night cycle (2–6). Deciphering the multicellular functioning of the SCN will, thus constitute a milestone on the way from clock genes to complex, overt rhythms.

Despite a high degree of coordination, SCN neurons are not always perfectly in phase. Stereotyped circadian waves in clock gene expression, intracellular calcium concentration (Ca^2+^_i_), and spontaneous firing rate of action potentials outline an approximate 6-hour phase gradient throughout SCN slices (3, 7–10). However, recent evidences challenged the rhythmic unity in the SCN, with some neurons exhibiting extreme phase lag. Phaseoids were defined as groups of neurons expressing the circadian clock protein PERIOD2 stably out of phase with their neighbors (11). Similarly, some SCN neurons appear electrophysiologically more active during nighttime, in total opposition of phase relative to the ensemble rhythm (12, 13). Last but not least, the circadian amplitude in multiunit electrophysiological activity is dramatically downsized in vivo as compared to in vitro preparations (14), suggesting even reduced neuronal synchronicity in living animals. How such a diversity in neuronal activity in vivo articulates within a coherent SCN network remains unclear.

Typically, fast Ca^2+^_i_ spikes driven by action potentials provide a fairly reliable estimate of neuronal activity. To date, variations in Ca^2+^_i_ have been extensively described at the circadian timescale in individual SCN neurons ex vivo (7, 8, 15–17) and at a neuronal population level in vivo (18, 19), but the spatiotemporal organization of SCN Ca^2+^_i_ spikes has been barely investigated, possibly because of technical issues with BAPTA-based Ca^2+^_i_ probes (20, 21). Here, we performed microendoscopic imaging of the Ca^2+^_i_ indicator GCamp6f at a single-cell resolution through a graded-index (GRIN) lens aimed at the SCN of freely behaving mice. Fast Ca^2+^_i_ spikes revealed unexpected diversity in daily neuronal activity patterns, and circuit-level signaling all around the light–dark cycle. Yet, we found that this diversity builds upon a highly coordinated wave in baseline Ca^2+^_i_ levels in the same cells. Together, Ca^2+^_i_ spikes and baseline Ca^2+^_i_ levels were independently modulated with the time of day in vivo, and reconciled the apparent discrepancy between functional cell diversity and network unity in the SCN.

## Results

**Fig. 1.**
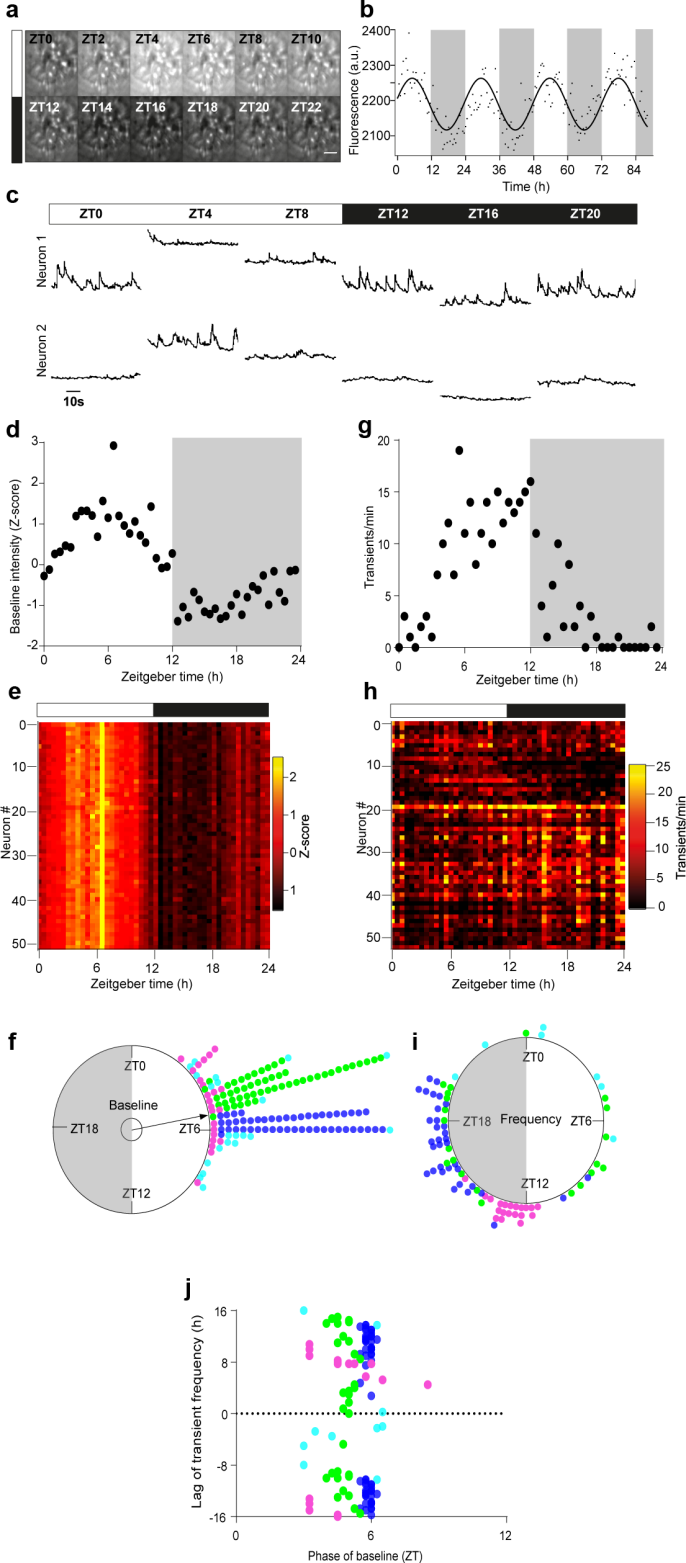
Daily diversity and unity of two Ca^2+^_i_ dynamics in individual SCN neurons in vivo. (a) Time-lapse projection of GCamp6f fluorescence monitored every 2 hours from zeitgeber time ZT = 0 to ZT = 22, through a GRIN lens aimed at the SCN of a freely moving mouse. Scale bar, 100 µm. (b) Daily variations in global GCamp6f signal intensity from one representative SCN field of view. The solid line represents the best cosine fit of experimental data points, measured every 30 minutes over four consecutive day–night cycles. (c) Longitudinal 1-minute recordings of GCamp6f fluorescence over 1 day–night cycle, from two SCN neurons within the same field of view. (d)–(i) Daily variation in GCamp6f baseline (d)–(f) and spike frequency (g)–(i), in SCN neurons. Example from one representative neuron (d) and (g), heat maps obtained from 52 neurons recorded in the same SCN field of view (e) and (h), and Rayleigh plots of significant individual daily peak phases measured from four different mice (f) and (i). Each color represents neurons from one mouse, examples were selected from the mouse color-coded in green. The inner circle represents the statistical threshold (*P* < 0.05) for the mean vector of the circular distribution of the aggregate data. The white and black boxes depict the light and dark phase, respectively. (j) Phase–lag relationship between spike frequency and the GCamp6f baseline.

After surgical preparation, mice were trained to the imaging setup for at least 3 weeks. They exhibited low levels of circulating corticosterone during their resting phase, which increased at the beginning of their active phase (Figure S1, Supplementary Material). Moreover, longitudinal monitoring of the global GCamp6f signal from the SCN (*n* = 12 mice) revealed daily changes, with peak and nadir in the middle of the light and dark phase (Fig. 1a and b), respectively, resembling the typical Ca^2+^_i_ rhythm recorded at the neuron population level using in vivo fiber photometry (18, 19). Hence, GRIN lens-implanted mice were free of chronic stress and maintained normal daily physiology under our experimental conditions.

At the individual cell level, two dynamics in GCamp6f intensity emerged from SCN neurons recorded in vivo, consisting in short-lived spikes (Movie 1) superimposed upon slow-evolving changes in basal fluorescence (Fig. 1c). To assess how these slow and fast dynamics evolved with the time of day, we monitored GCamp6f fluctuations during 1-minute episodes, every 30 minutes. We were able to follow 155 individual SCN neurons longitudinally over at least 24 hours, from four different mice. The baseline GCamp6f level displayed a significant daily pattern in up to 145 neurons (70% to 100%), as assessed by nonparametric JTK-Cycle analysis (22). In every rhythmic neuron, the baseline level reached its maximum during daytime (Fig. 1d–f; Figure S2, Supplementary Material), around zeitgeber time ZT = 6 (with ZT = 0 at lights on, 95% CI [ZT = 03:28, ZT = 07:11], *P* < 0.001 Moore’s modified Rayleigh test). The interindividual variability between all four mouse SCN (*P* < 10^–7^, F(3, 141), Watson–Williams F-test) suggested subtle topographical heterogeneity that was reminiscent of the phase gradient in circadian Ca^2+^_i_ rhythm observed in SCN slices (7, 8). Hence, the daily variations in baseline Ca^2+^_i_ levels underscored the unity of individual neuronal oscillators in the SCN in vivo.

By contrast, fast GCamp6f spikes from the same neurons could occur at all times of day, and revealed the diversity in daily Ca^2+^_i_ activity patterns in the SCN. A total of 38% to 69% of the recorded neurons exhibited a significant daily organization in spike frequency (Fig. 1g–i; Figure S3, Supplementary Material). In two out of four mice, most neurons had their peak activity phase at the light–dark transition or in the early night. In the other two mice, the peak phase was more widely distributed. This resulted in a globally large phase dispersion of fast spikes (*P* > 0.1 Moore’s modified Rayleigh test, Fig. 1i), and a stratified phase–lag relationship with the daily rhythm in baseline levels (Fig. 1j), which indicated that fast Ca^2+^_i_ spike activity and basal Ca^2+^_i_ levels in SCN neurons were independently modulated with the time of day.

**Fig. 2.**
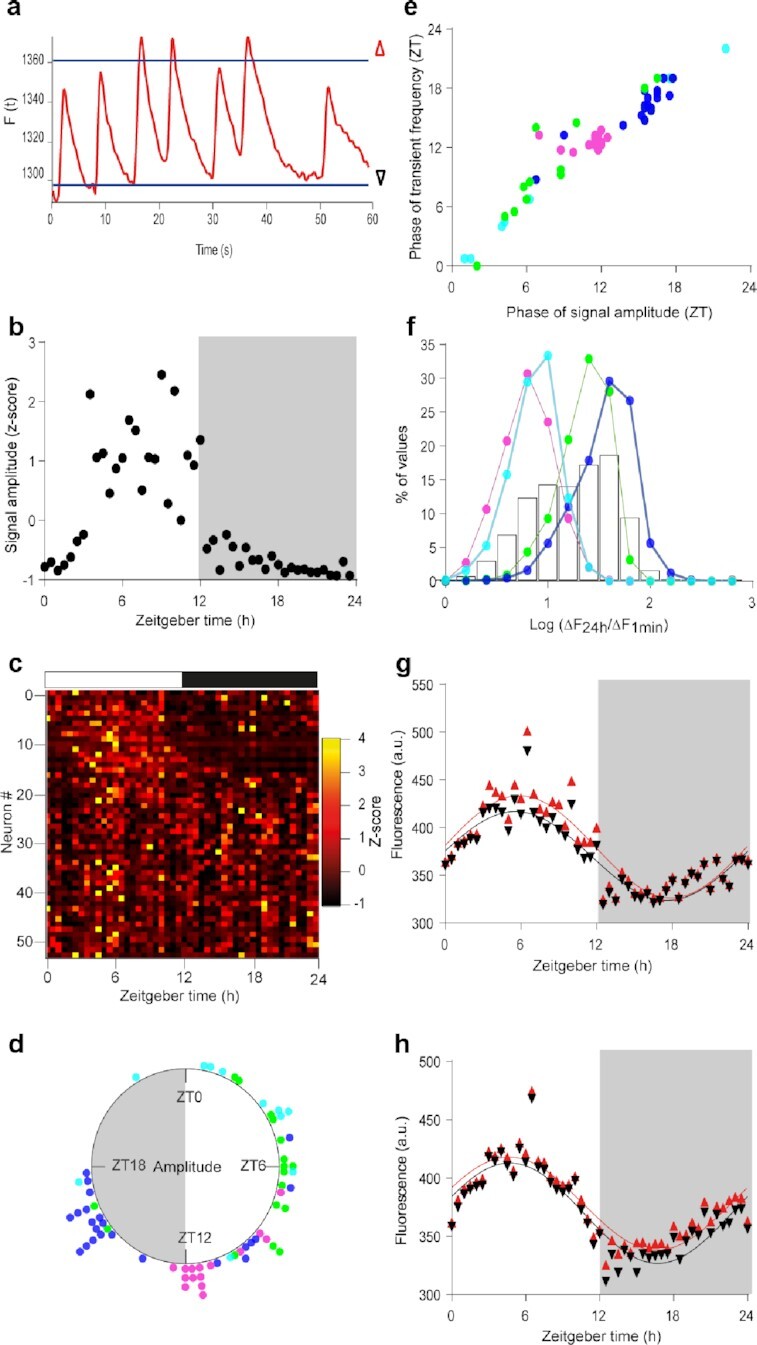
Small Ca^2+^_i_ spikes built upon large daily changes in basal Ca^2+^_i_ levels. (a) 1-minute recording of GCamp6f fluorescence, with thresholds for the 5th and 95th percentiles (baseline and topline, black and red triangles, respectively) used to calculate the signal amplitude. (b)–(d) Daily variations in GCamp6f signal amplitude in the same neuron (b) and same SCN field (c) as in Fig. 1. (d) Rayleigh plot of significant individual daily peak phases in signal amplitude for the aggregate data. (e) Phase relationship between spike frequency and signal amplitude. (f) Distribution of the ratio values calculated, for each 1-minute recording, between the daily amplitude in baseline GCamp6f (ΔF_24h_) and the signal amplitude over 1 minute (ΔF_1min_), for each mouse SCN (colored lines) and the aggregate data (black bars). (g) and (h) Daily profiles in GCamp6f baseline and topline (as depicted in a) from two representative SCN neurons, with larger signal amplitude during the day (g) or night (h). The solid lines represent the best cosine wave for each dataset.

To gain insight into the contribution of spikes to the daily Ca^2+^_i_ rhythm in SCN neurons, relatively to baseline changes, we measured the amplitude of changes in GCamp6f fluorescence in each 1-minute recording (Fig. 2a). This parameter followed a daily organization in about half (33% to 52%) of the recorded neurons (Fig. 2b–d; Figure S4, Supplementary Material), roughly phase-locked to spike frequency (Spearman r = 0.94, *P* < 0.0001; Fig. 2e; Figure S5, Supplementary Material). Spiking activity was generally one-to-two orders of magnitude smaller than the variation in basal GCamp6f fluorescence measured over 24 hours in the same neuron (Fig. 2f). Therefore, spikes contributed moderately to the daily rhythmicity in fluorescence, regardless of whether they occurred at high or low baseline levels (Fig. 2g and h). Together, our data indicated that slow changes in basal Ca^2+^_i_ levels and fast Ca^2+^_i_ spikes were nested dynamics that respectively supported the daily rhythm unity and functional diversity in the SCN cell network in vivo.

**Fig. 3.**
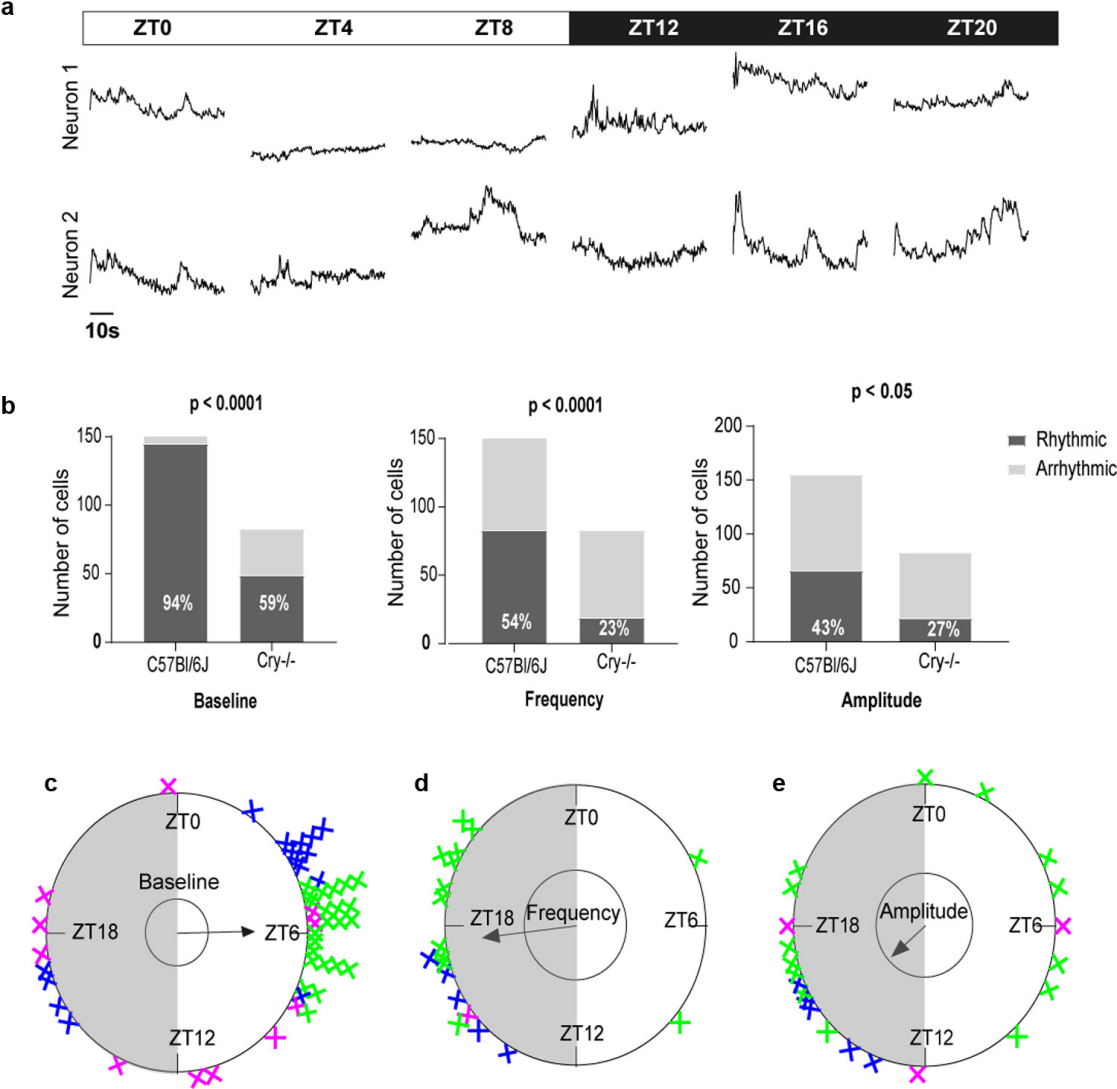
Daily variations in slow and fast Ca^2+^_i_ dynamics in *Cry1-/- Cry2-/-* mice. (a) Longitudinal 1-minute recordings of GCamp6f fluorescence over 1 day–night cycle, from two SCN neurons of one *Cry1-/- Cry2-/-* mouse. (b) Quantitation of SCN neurons exhibiting a significant daily rhythm in GCamp6f baseline level (left), spike frequency (middle), and signal amplitude (right), in C57Bl/6 J and *Cry1-/- Cry2-/-* mice. The *P-*values indicate a significant genotype-dependence in the proportion of rhythmic neurons for each parameter (Fisher's exact contingency test). (c)–(e) Rayleigh plots of significant individual daily peak phases in baseline level (c), spike frequency (d), and signal amplitude (e) measured from three *Cry1-/- Cry2-/-* mice. Each color represents neurons from one mouse, examples in (a) were selected from the mouse color-coded in blue.

How much is the circadian clock responsible for the daily organization of each of these Ca^2+^_i_ dynamics? To address this issue, we conducted an independent set of GCamp6f measurements in the SCN of globally clockless *Cry1-/- Cry2-/-* mutant mice (23). Longitudinal recordings of 83 individual neurons from three mice revealed conserved fast spikes and slow changes in GCamp6f baseline (Fig. 3a), but with a disturbed daily distribution. *Cry1-/- Cry2-/-* mice are known to maintain a marked alternation of diurnal rest and nocturnal locomotion under normal light–dark conditions such as those used in our study (23, 24). However, the proportion of neurons exhibiting a significant daily organization in GCamp6f signals was reduced by about half in absence of a functional circadian clock as compared to control mice, for both the basal level and the frequency or amplitude of fast spikes (Fig. 3b–e; Figure S2–S4, Supplementary Material). Moreover, although a majority of *Cry1-/- Cry2-/-* SCN neurons still reached their maximal baseline level during daytime, the overall peak phase distribution was wider than for control mice, with a number of SCN neurons peaking at night in two out of three mice (*P* > 0.1 Moore’s modified Rayleigh test, Fig. 3e; Figure S2, Supplementary Material). These results indicated that the daily organization of both slow basal Ca^2+^_i_ changes and fast Ca^2+^_i_ spikes depended on a functional circadian clock.

**Fig. 4.**
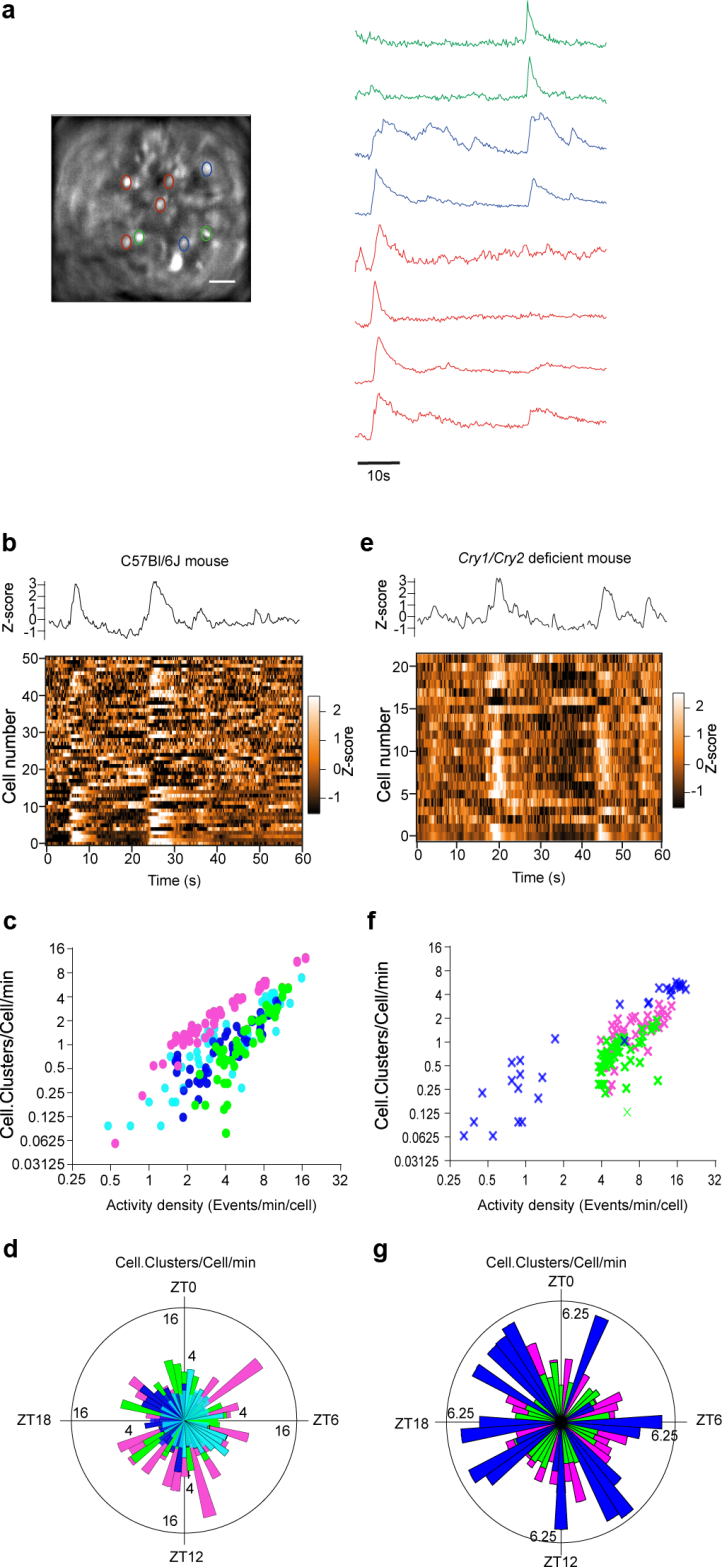
Synchronous fast Ca^2+^_i_ spikes depicted clock-independent cell–cell signaling in the SCN. (a) 1-minute recordings of GCamp6f fluorescence from SCN neurons in a same field of view. Note the occurrence of synchronous spikes in neuron subsets (delineated by three different colors). Scale bar, 100 µm. (b)–(g) Analysis of clusters of coactivity from the SCN of C57Bl/6 J (b)–(d) and *Cry1-/- Cry2-/-* (e)–(g) mice. (b) and (e) Global GCamp6f fluorescence (upper panel) and heat map of signal intensity from individual neurons (lower panel), recorded during 1 minute. (c), (d), (f), and (g) Extent of clusters of coactivity as a function of the density in activity (c) and (f) and time of day (d) and (g).

Next, we explored the spatiotemporal organization of GCamp6f spikes at the multicellular scale to question the circuit-level significance of the fast Ca^2+^_i_ activity in the SCN. We found that fast GCamp6f spikes could occur within the same time frame in clusters of SCN neurons that partially overlapped each other and involved enough neurons to generate noticeable changes in the global field signal (Fig. 4a and b; Movie 1). The extent of coactivity clusters in the field of view, as expressed in cell.clusters/cell/min, correlated positively to the density of neuronal activity (Spearman r = 0.71, *P* < 0.0001, Fig. 4c). Since we computed coactivity only when detected above chance threshold (see methods), this correlation between coactivity and activity density could not result solely from random coincidence of Ca^2+^_i_ spikes in highly active neurons. More likely, the clusters of coactivity depicted circuit-level Ca^2+^_i_ signaling in the SCN (7).

Accordingly, the extent of coactivity was maximum in each mouse when most neurons reached their peak in activity (Figures S3 and S6, Supplementary Material). If one considered data from all four mice globally, coactivity spread evenly around the clock (*P* > 0.5, Moore’s modified Rayleigh test, Fig. 4d), suggesting that synchronizing cues circulated across the SCN cell network in vivo at all times of the light–dark cycle. Moreover, coactivity persisted under the conditions of an altered circadian clock, both during jetlag, when the global GCamp6f rhythm was progressively resetting to a new light phase (*n* = 3 mice, Figure S7, Supplementary Material), and in *Cry1-/- Cry2-/-* mice, in a similar relationship with activity density as in control mice (Spearman r = 0.88, *P* < 0.0001, Fig. 4e–g). Together, these data revealed extensive cell–cell coordination independent from the circadian clock in the SCN of free-behaving mice.

## Discussion

In this study, we used in vivo Ca^2+^_i_ imaging to investigate spatiotemporal Ca^2+^_i_ signaling at a single-cell resolution in the SCN of free-behaving mice. We found that daily Ca^2+^_i_ rhythmicity in SCN neurons is composed of fast Ca^2+^_i_ spikes superimposed upon slow variations in baseline Ca^2+^_i_ levels. Similarly, such slow and fast Ca^2+^_i_ dynamics were recently reported in circadian pacemaker neurons of Drosophila (25). Altogether, these results reveal conserved properties between Invertebrates and Vertebrates in the cell biology of daily Ca^2+^_i_ rhythms in circadian timekeeping systems.

In mouse SCN neurons as well as in Drosophila clock neurons, fast Ca^2+^_i_ spikes underscored a wide array in daily activity patterns. This diversity was obvious between the SCN from different mice, but also between neurons from the same individual, suggesting a topographical distribution of activity patterns, like in Drosophila. Overall, a majority of SCN neurons were more active at night. As Ca^2+^_i_ spikes are typically associated with electrophysiological activity (21, 25), this observation may first appear surprising in view of the many evidences of daytime-biased firing of action potentials in the SCN (3–5, 14, 26). Yet, it is actually in line with the high firing rate measured during nighttime from the SCN of live animals as compared to slice preparations (14), and suggests a widespread occurrence in vivo of the night-active neuron populations reported in SCN slices (12, 13).

Unlike in Drosophila clock neurons (25), SCN rhythms in fast Ca^2+^_i_ activity and basal Ca^2+^_i_ levels are not cophasic. Daily variations in basal Ca^2+^_i_ levels reach their peak phase around midday in all the recorded SCN neurons. This homogeneity contrasts with the diversity in fast Ca^2+^_i_ activity patterns, and depicts the global unity of individual neuron rhythmicity within the SCN cell network assumed to confer robustness and precision to the mammalian circadian pacemaker (4–6).

Since slow and fast Ca^2+^_i_ dynamics are independently modulated with the time of day in the mouse SCN, they provide an additional level of combination as compared to Drosophila clock neurons. The occurrence of small and fast Ca^2+^_i_ spikes at various time points upon the large and slow change in baseline extents the repertoire of Ca^2+^_i_ signaling in the SCN, from the typical binary alternation of up and down states, toward the possibility of an activity-dependent multiplex code. It is then tempting to speculate that neurons exhibiting fast Ca^2+^_i_ spikes during e.g. their down Ca^2+^_i_ state may contribute in different functions than neurons active during their up state. Conversely, neurons using one same Ca^2+^_i_ code might constitute functional units, gating specific body rhythms (13, 27–30). The composite tuning of Ca^2+^_i_-dependent downstream effectors identified in SCN neurons, such as the multisite phosphorylation of CREB (31, 32), affords a possible molecular mechanism to decipher this Ca^2+^_i_ code.

One limitation of our study is that we could not locate precisely the recorded cells, nor establish whether the diversity in activity patterns corresponds to the diversity in peptidergic content among SCN neurons (33–37). Topographical differences in the timing and origin of Ca^2+^_i_ signaling have been reported in SCN slices (7, 8, 38). As we implanted GRIN lenses dorsally to the SCN in order not to lesion circadian neurons, we assume that we performed recordings in the shell SCN, enriched in vasopressin-expressing neurons. The slow entrainment of the GCamp6f rhythm during simulated jetlag (Figure S7b, Supplementary Material) is in line with this assumption as retino-recipient VIP-containing neurons are expected to phase-shift rapidly (19). However, the AAV-driven expression of GCamp6f under a nonspecific promoter does not allow the assignment of activity patterns to identified neuronal subtypes. Further experiments targeting either SCN neuronal population (39) will be needed to fill this gap.

At the multicellular level, fast Ca^2+^_i_ spikes revealed evidence of circuit-level communication. Synchronous events occurred at all times of day and persisted under circadian clock disruption, in line with clock-independent network properties in the SCN (10, 40). Hence, fast Ca^2+^_i_ spikes are both hints of individual neuron activity, and mediate intercellular signaling, indicating that the mammalian circadian pacemaker ticks also at the timescale of seconds. As individuating part-whole relations in neural circuits has become an important challenge (41, 42), our study illustrates how the combination of slow and fast Ca^2+^_i_ dynamics in the SCN of free-behaving mice supports the diversity in individual neuron activity patterns within the temporal unity of the global network.

## Materials and methods

### Animals

All mouse experiments complied with the European Directive 2010/63/UE, and were registered under the reference APAFIS#15,032–2018050918181227 v2. Male C57Bl/6 J mice (5 to 6 weeks old) were purchased from Janvier Labs (Le Genest-Saint-Isle, France). *Cry1-/- Cry2-/-* mice (23) were produced by breeding double-heterozygous males and females from our colony maintained in a C57Bl6/J background for more than 20 generations (43). All mice were housed in ventilated microisolator cages, under a 12 h:12 h light:dark cycle, with free access to food and water. For simulated jetlag experiments, the light–dark cycle was advanced by 6 hours, starting with the shift of light onset.

### Surgery

At the age of 8 weeks, mice were anesthetized with a cocktail of Ketamine (Imalgene 500, 75 mg/kg) and Xylazine (Rompun, 10 mg/kg). A skin incision exposed the skull, and small craniotomies were made dorsal to the injection site with a drill mounted on a stereotaxic apparatus. The virus solution (1,000 nl, titer ≥ 1,013 vg/ml, #100836-AAV9 from Addgene) was injected in the SCN (ML +/−0.2, AP 0, and DV −5.7) at a rate of 50 nl/minute using a microinjector-controlled syringe (micro-4, World Precision Instruments) and needle (Nanofil 33 G beveled needle, World Precision Instruments). The needle was kept in place for 10 minutes following injection to allow suitable diffusion, and reduce backflow during withdraw. Mice were then implanted with a GRIN lens (diameter 0.6 mm, length 7.44 mm, and working distance 150 µm, #AB000436 from GRINTECH), aimed 50 to 100 µm dorsally to the virus injection site. The lens was stabilized with dental cement (Metabond) and Kwik-Silsealant (World Precision Instruments). After 3 to 4 weeks, mice were anesthetized for placement of the microendoscope baseplate (#1050–004638, Inscopix), and a baseplate cover (#1050–004639, Inscopix) was used to protect the lens when not in use.

After surgery, mice were housed in individual cages, and regularly habituated to the imaging setup, with a dummy microendoscope (DMS-2, Inscopix) for at least 3 weeks. To ascertain well-being and normal daily physiology under these conditions, tail-tip blood (6 µl) was collected from four mice at ZT7 and ZT12 to check corticosterone levels (ELISA kit From Assaypro).

### Microendoscopic data acquisition and processing

Images were acquired with a head-mounted miniaturized microscope (nVista, Inscopix) at 4 frames per second over 1 minute (240 ms exposure time, 10% to 20% LED illumination, 1.5 to 2.5x gain). The 1-minute movies were spatially filtered (high-pass filter, cutoff at 40 µm), corrected for motion artifacts (Inscopix Data Processing Software), and saved as stacks of frames in TIFF format. Regions of interest (ROI) were defined as nonoverlapping bright regions that displayed fast activity in any stack. They were applied to every stack (ImageJ), and numerical data were saved as text files for further processing in Matlab (MathWorks).

### Data analysis and statistics

A Butterworth low-pass filter was applied (cutoff frequency at 1.1 Hz). After detrending, the 5th and 95th values were computed for each 1-minute recording. The 5th percentile value was considered as the baseline level, and the difference between the 5th and 95th values defined the signal amplitude. Fast spikes were detected as fast and large events with a two-threshold procedure. Respectively, threshold 1 selected the fastest events from all recordings (90th percentile of all derivative values from all recordings, from all ROIs), and threshold 2 eliminated the smallest of these events for each recorded neuron (set at 2 SDs of all the raw signals over the course of a 24-hour period for each ROI). For each recording, the spike frequency was calculated as the number of spikes detected during 1 minute. The activity density was computed as the spike frequency divided by the number of recorded cells in the field of view.

The onset of spikes (derivative crossing above 0) was computed as their time of occurrence. Ca^2+^_i_ spikes occurring in a high-activity frame in a statistically significant number of neurons delineated the clusters of coactivity. The threshold corresponding to a significance level of *P* < 0.05 was defined as the number of activated cells in a single frame that exceeded only 5% of 500 surrogate datasets obtained by randomly transposing intervals of activity within each cell (44). The extent of clusters of coactivity was computed as the sum of all the cells participating in any of the coactive events during a 1-minute recording, normalized by the total number of cells recorded in the field (expressed in Cell.Clusters/Cell/min).

Nonparametric JTK-Cycle analysis (22) was used to estimate the daily phase of the GCamp6f signal parameters from datasets recorded over one 24-hour period. Circular analysis and statistics were conducted using Oriana (Kovach Computing Services). Other numerical and statistical analyses were conducted with IgorPro (WaveMetrics, Inc), Prism 7 (GraphPad Software, Inc).

## Supplementary Material

pgac112_Supplemental_Files

## Data Availability

All data is included in the manuscript and/or supporting information.
