# Supplementary material for: Nested calcium dynamics support daily cell unity and diversity in the suprachiasmatic nuclei of free-behaving mice
Source: PNAS Nexus. 2022 Jul 11;1(3):pgac112. doi: 10.1093/pnasnexus/pgac112 (PMC9896879; doi:10.1093/pnasnexus/pgac112)
Supplement: pgac112_Supplemental_Files [file pgac112_supplemental_files.zip › PNASNEXUS-PNASNEXUS-2022-00175-T-s02.pdf]

Supplementary Information for
Nested calcium dynamics support daily cell unity and diversity in the suprachiasmatic nuclei of free-behaving mice

Lama El Cheikh Hussein, Pierre Fontanaud, Patrice Mollard and Xavier Bonnefont.

Xavier Bonnefont
Email: Xavier.Bonnefont@igf.cnrs.fr

This PDF file includes:

Figures S1 to S7
Legend for Movie S1

Other supplementary materials for this manuscript include the following:

Movie S1

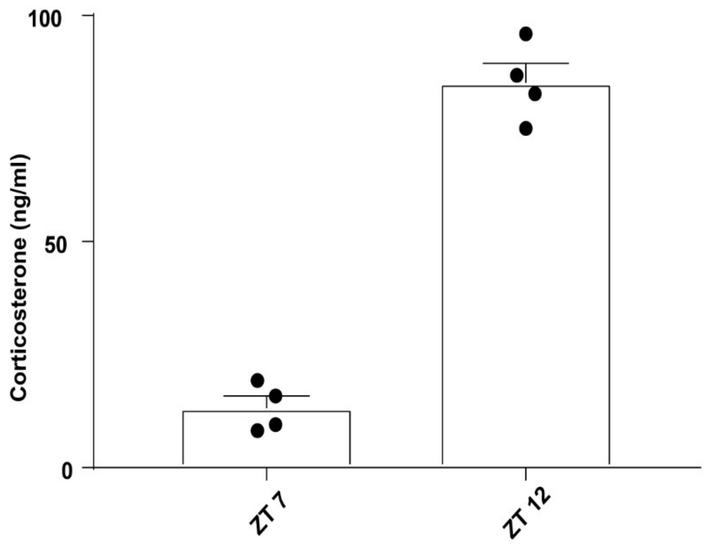

Fig. S1. : Corticosterone concentration in the blood of mice implanted with a GRIN lens and equipped with the miniscope. Note the low level of stress, and the physiological increase in blood corticosterone around the onset of lights off (ZT12). Mean + s.e.m., n=4.

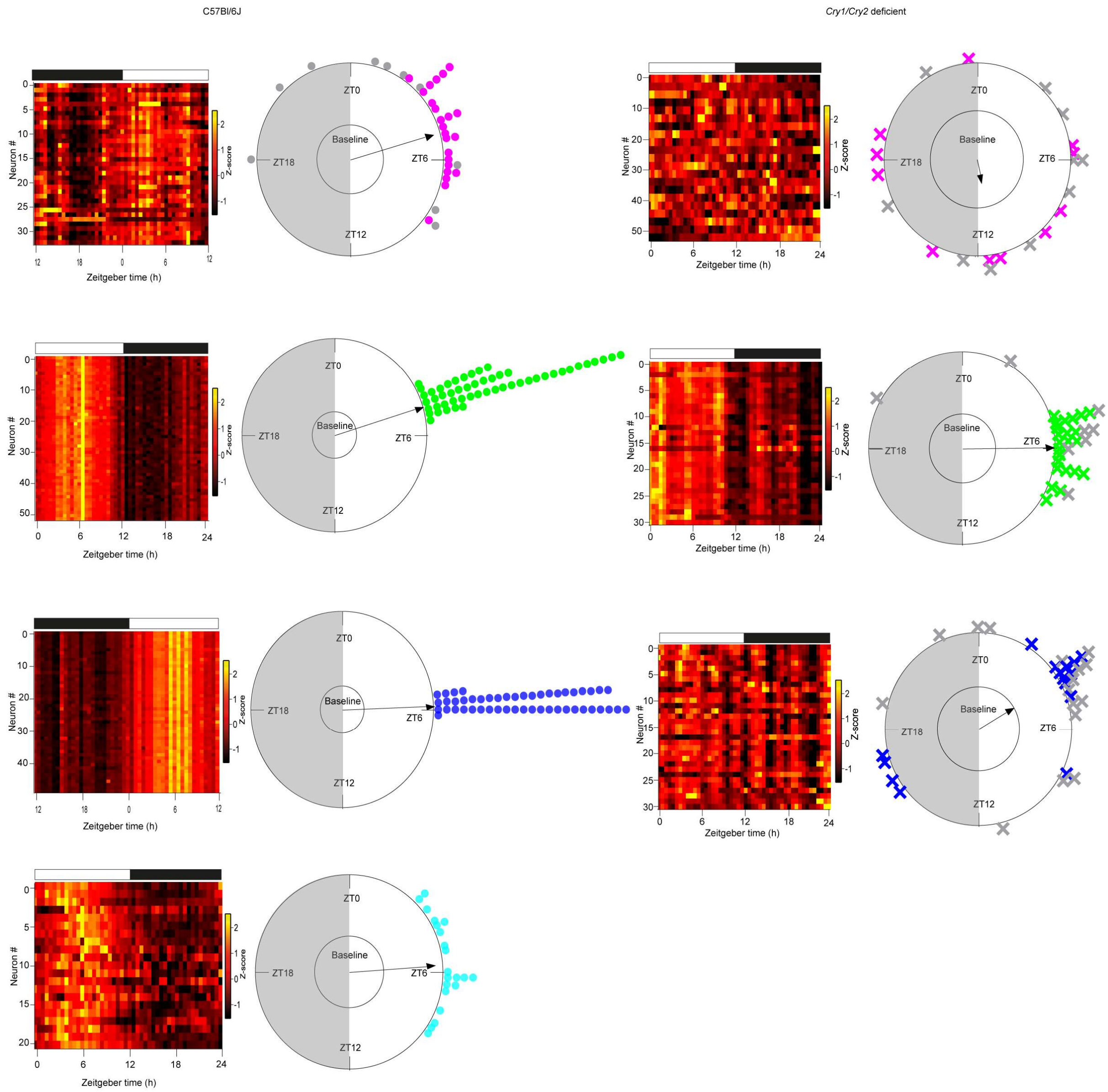

Fig. S2. Baseline Ca^{2+}_i levels from individual datasets. Heat maps depicting the longitudinal variation in basal Ca^{2+}_i levels, and Rayleigh plots of the daily peak phases, from C57Bl/6J **(left)** and *Cry1-/- Cry2-/-* **(right)** mice. Colored symbols represent neurons with a significantly daily rhythmic pattern (as assessed by JTK-Cycle). Non-rhythmic neurons are depicted by the grey symbols. The inner circle represents the statistical threshold ($p<0.05$) for the mean vector of the circular distribution of significantly rhythmic neurons only.

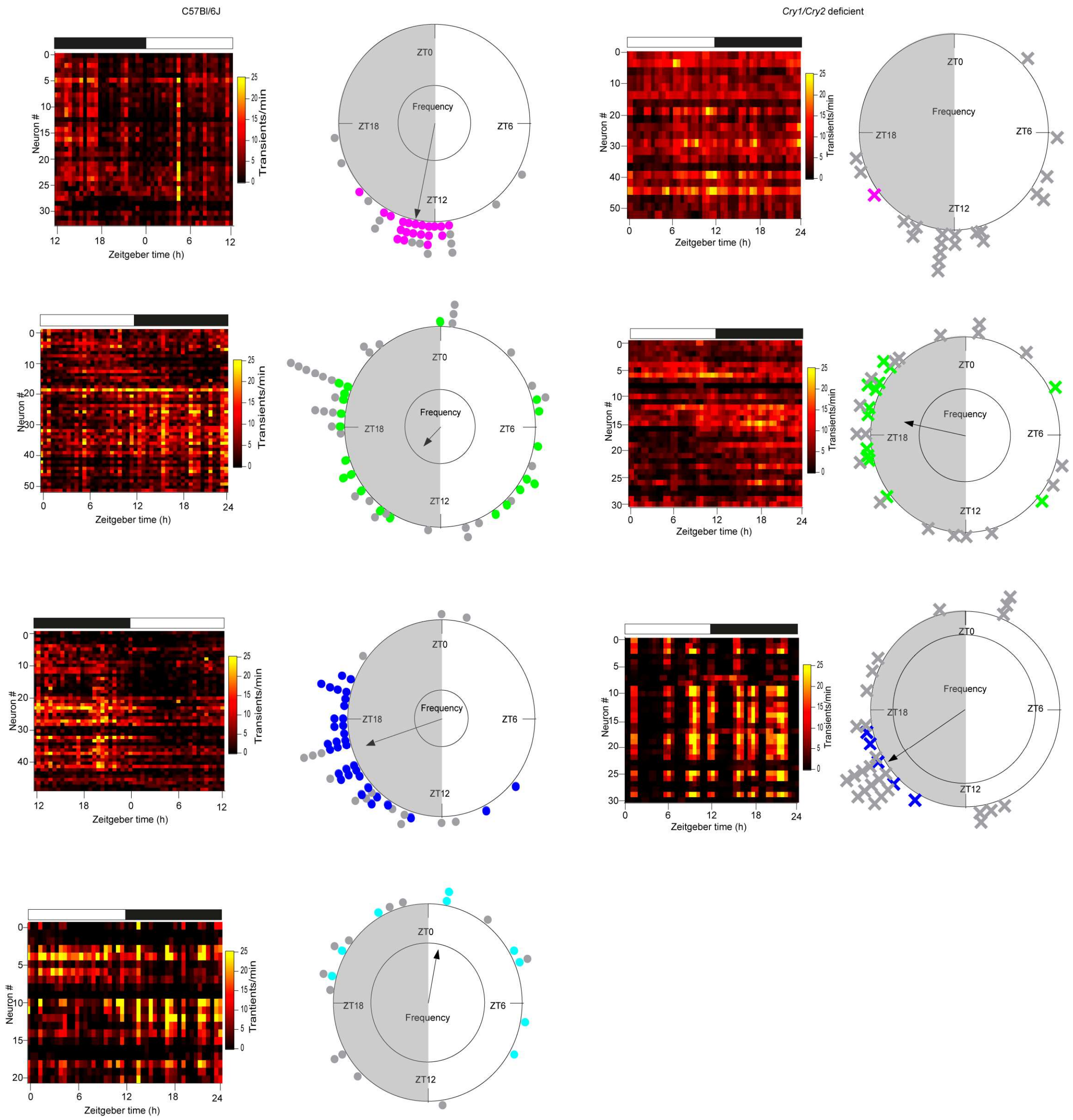

Fig. S3. Frequency of fast Ca^{2+}_i transients from individual datasets. Heat maps depicting the longitudinal variation of Ca^{2+}_i transient frequency, and Rayleigh plots of the daily peak phases, from C57Bl/6J **(left)** and *Cry1-/- Cry2-/-* **(right)** mice. Colored symbols represent neurons with a significantly daily rhythmic pattern (as assessed by JTK-Cycle). Non-rhythmic neurons are depicted by the grey symbols. The inner circle represents the statistical threshold ($p<0.05$) for the mean vector of the circular distribution of significantly rhythmic neurons only.

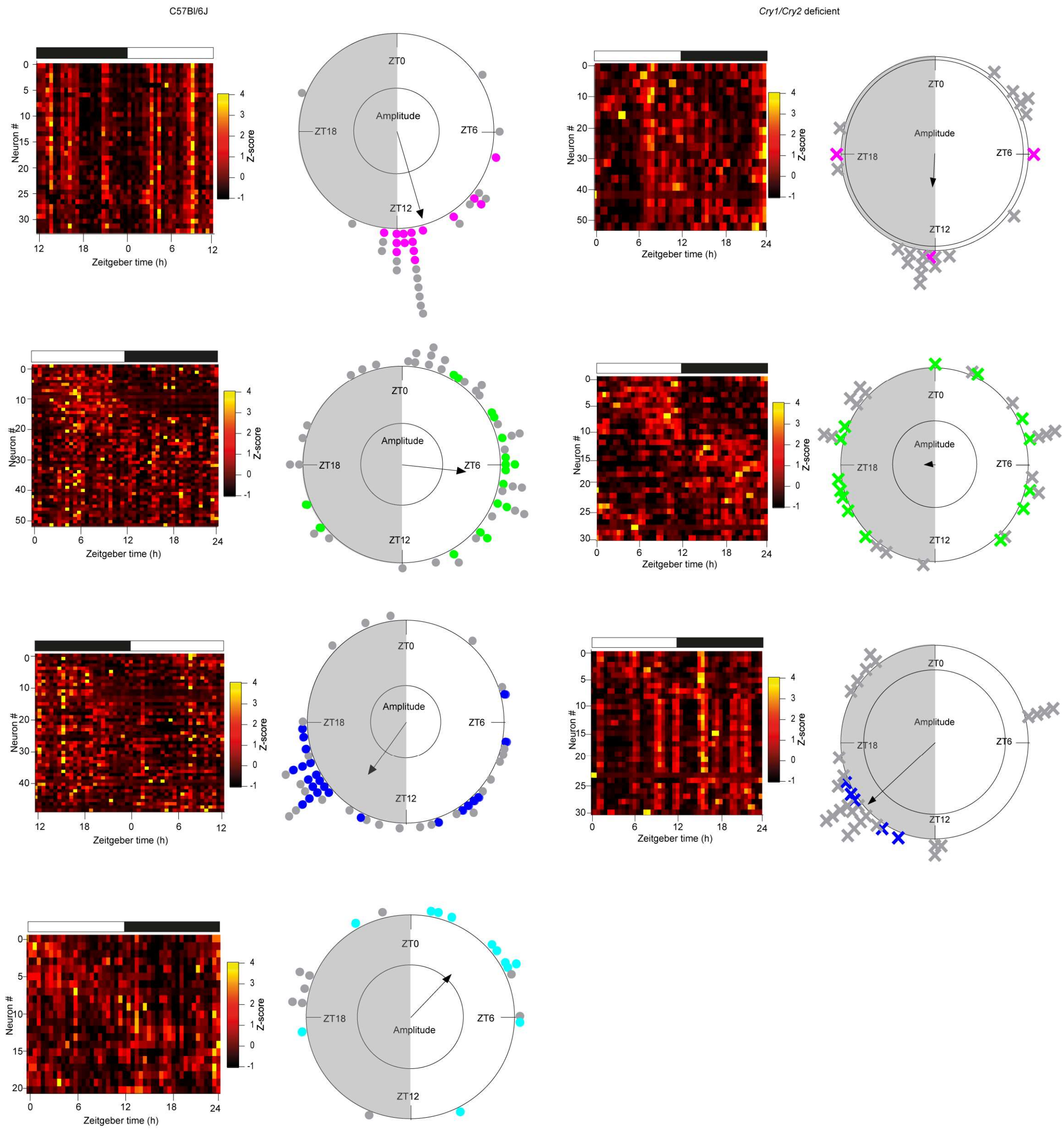

Fig. S4. Amplitude of fast Ca$^{2+}_{i}$ activity in individual datasets. Heat maps depicting the longitudinal variation of GCamp6f signal amplitude, and Rayleigh plots of the daily peak phases, from C57Bl/6J **(left)** and *Cry1-/- Cry2-/-* **(right)** mice. Colored symbols represent neurons with a significantly daily rhythmic pattern (as assessed by JTK-Cycle). Non-rhythmic neurons are depicted by the grey symbols. The inner circle represents the statistical threshold (p<0.05) for the mean vector of the circular distribution of significantly rhythmic neurons only.

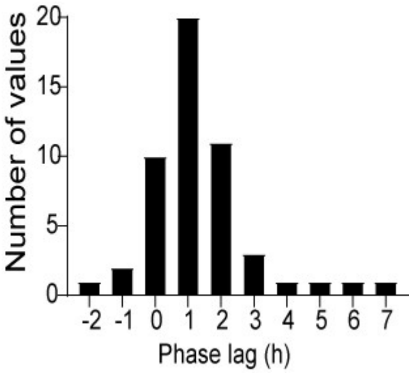

Fig. S5. Phase lag between signal amplitude and spike frequency.

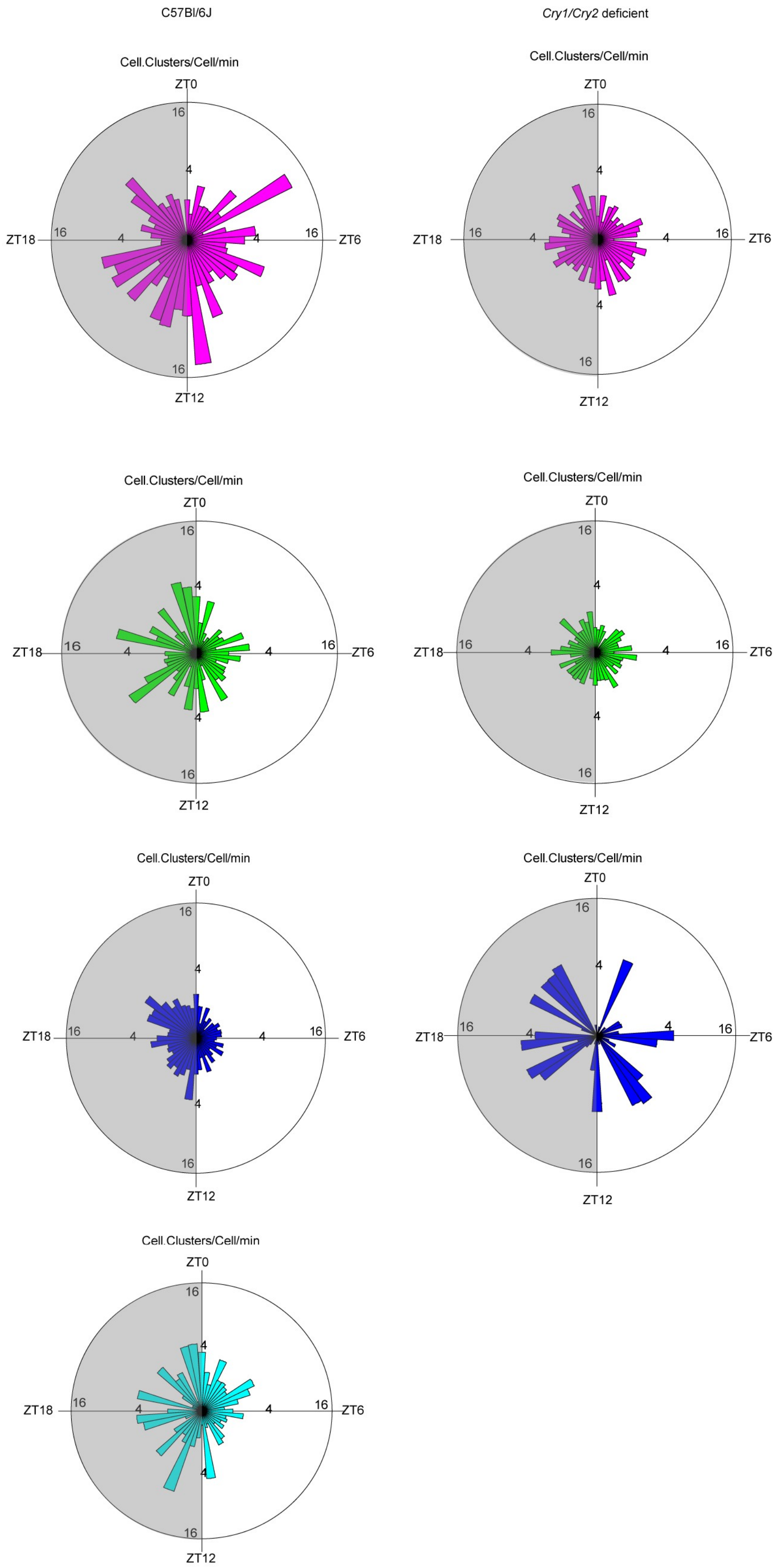
C57Bl/6J
Cry1/Cry2 deficient
Cell.Clusters/Cell/min
ZT0
ZT6
ZT12
ZT18
16
4

Fig. S6. Circular phase-distributions of clusters in fast Ca^{2+}_i co-activity from individual datasets. The plots depict the extent of coactivity clusters as a function of time in the SCN of C57Bl/6J **(Left)** and *Cry-/- Cry2-/-* **(Right)** mice.

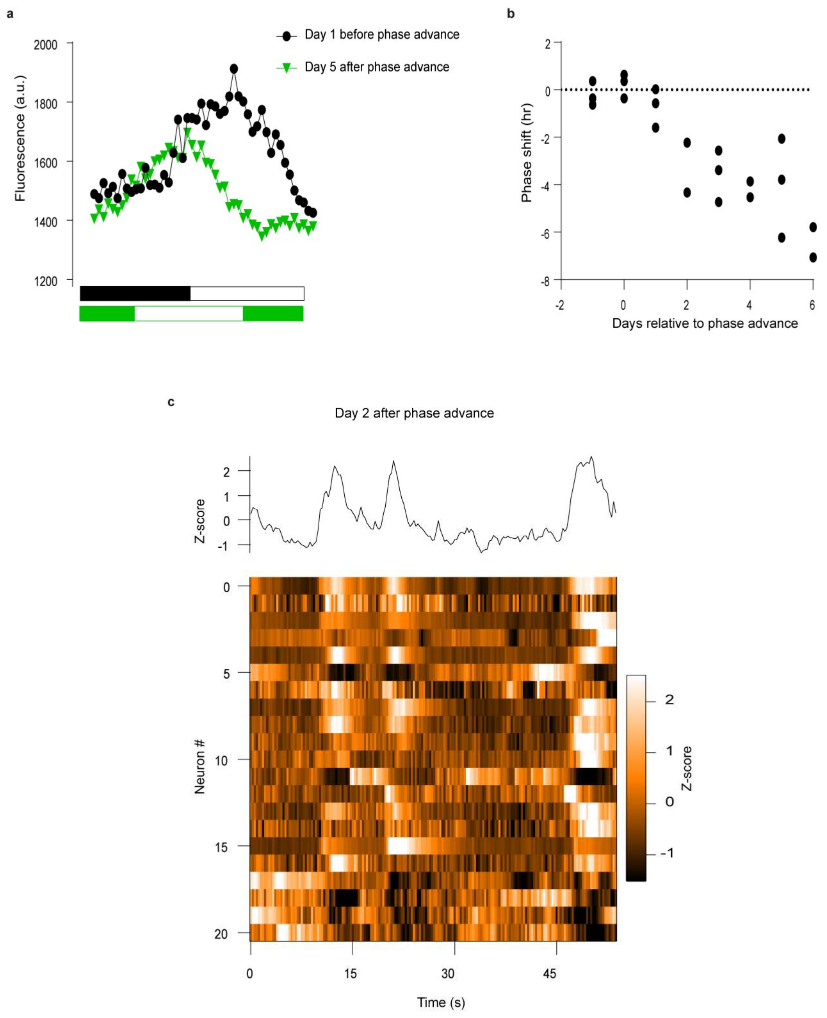

Fig. S7. ***In vivo* monitoring of GCamp6f in the mouse SCN during jetlag. a,** Daily variations in global GCamp6f fluorescence in one mouse, before (in black) and 5 days after (in green) a 6-hour advance of the light cycle. The white and colored boxes depict the light and phase during each period, respectively. **b,** Phase entrainment of GCamp6f fluorescence in the SCN during jetlag. The phase angle was estimated at the time of minimal fluorescence intensity (n=2-3 mice for each cycle). **c,** Global GCamp6f fluorescence (upper panel) and heat map of signal intensity from individual neurons (lower panel) recorded during the transition period, two days after the light phase shift.

Movie S1 (separate file). Microendoscopic recording of GCamp6f fluorescence in a mouse SCN *in vivo*. This movie represents a 20-minute recording session, at 4 frames per seconds (accelerated 62.5 times here).
